# Perspectives in Intraoperative Diagnostics of Human Gliomas

**DOI:** 10.1155/2015/479014

**Published:** 2015-10-12

**Authors:** O. Tyurikova, Y. Dembitskaya, K. Yashin, M. Mishchenko, M. Vedunova, I. Medyanik, V. Kazantsev

**Affiliations:** ^1^Lobachevsky State University of Nizhni Novgorod, Institute of Biology and Biomedicine, Russia; ^2^Federal State Budgetary Institution, “Privolzhsky Federal Research Medical Centre” of the Ministry of Health of the Russian Federation, Russia; ^3^Nizhny Novgorod State Medical Academy, Russia

## Abstract

Amongst large a variety of oncological diseases, malignant gliomas represent one of the most severe types of tumors. They are also the most common type of the brain tumors and account for over half of the astrocytic tumors. According to different sources, the average life expectancy of patients with various glioblastomas varies between 10 and 12 months and that of patients with anaplastic astrocytic tumors between 20 and 24 months. Therefore, studies of the physiology of transformed glial cells are critical for the development of treatment methods. Modern medical approaches offer complex procedures, including the microsurgical tumor removal, radiotherapy, and chemotherapy, supplemented with photodynamic therapy and immunotherapy. The most radical of them is surgical resection, which allows removing the largest part of the tumor, reduces the intracranial hypertension, and minimizes the degree of neurological deficit. However, complete removal of the tumor remains impossible. The main limitations are insufficient visualization of glioma boundaries, due to its infiltrative growth, and the necessity to preserve healthy tissue. This review is devoted to the description of advantages and disadvantages of modern intraoperative diagnostics of human gliomas and highlights potential perspectives for development of their treatment.

## 1. Introduction

Tumors located in the brain are specifically important targets for medical treatment due to their high severity and mortality rate as compared to many other oncology diseases. The average life expectancy of patients with brain tumors such as glioblastoma, according to different authors, is less than one year, and for patients with anaplastic astrocytic tumors from 20 to 24 months [1]. Tumor cells in the brain mainly develop from astrocytes. Thus, the most common type of the brain tumors, malignant gliomas, accounts for 63% of all astrocytes tumors [2]. According to the World Health Organization classification, based on the level of malignancy, tumors are divided into several grades: glioma I degree, which represents the initial stage of development of gliomas, and glioma II–IV degrees, which are malignant tumors that differ in terms of their activity [3–5]. The most aggressive glioblastoma grade is IVth degree, since the life expectancy of patients after treatment with this type of tumor is an average of 12 to 15 months [6]. The particular treatment of gliomas is defined by the level of malignancy, type of tumor, and age of patients. Understanding of the distinct physiology of tumor and normal cells is the key aspect for the development of treatments and diagnostics.

## 2. Physiological Comparison between Gliomas and Normal Glial Cells

**Figure 1:**
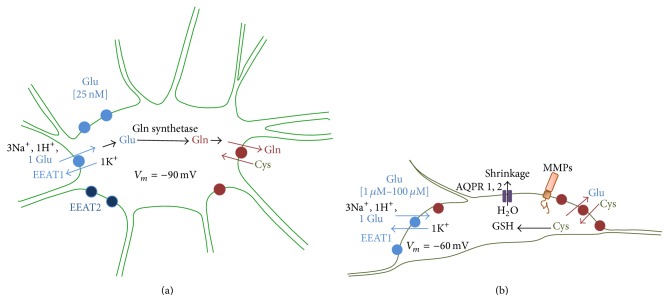
Physiological comparison of normal and reactive astrocytes. (a) Normal astrocytes show a high level of expression of EAATs 1 and 2, which control the extracellular glutamate concentration around 25 nM. The cysteine-glutamate antiporters (X^−^_*c*_) provide the exchange of cysteine from the extracellular space on glutamine. Transformation of glutamate (Glu) to inactive form, glutamine (Gln), is carried out by glutamine synthase. The resting membrane potential in normal astrocytes holds around −90 mV; (b) the expression of EAAT1 in reactive astrocytes is significantly lower than in normal, whereas the EAAT2 type is absent which leads to the increase of extracellular glutamate concentration up to 1–100 *μ*M. The cysteine-glutamate antiporters (X^−^_*c*_) in reactive astrocytes perform the exchange of cysteine from the extracellular space to glutamate which causes additional increase of extracellular glutamate concentration. Inside the astrocyte cystein (Cys) is converted to the glutation (GSH) and leads to the rise of resistance to oxidation. The resting membrane potential in reactive astrocytes equilibrates around −60 mV due to alterations in chloride homeostasis. Reactive astrocytes regulate their volume by releasing water through aquaporin channels (AQPR 1 and 2) and are characterized by a higher expression of metalloproteinases (MMTs), which break down the surrounding extracellular matrix and thus produce tunnels to the cell migration.

Glioma cells develop from different types of glial cells [7, 8]. Therefore, they have various common as well as distinct physiological characteristics (Figure 1). Specifically, glioma cells are characterized by disruptions in the glutamate maintaining, receptors and transporters expression. Additionally, they have alterations in the resting membrane potential and ion homeostasis which lead to their ability to migrate throughout the brain. Detailed studies and generalization of existing knowledge about the diversity in the physiology of gliomas and glial cells are especially important for the development of diagnostics.

### 2.1. Maintaining Glutamate Concentration

One of the key differences between normal and reactive astrocytes in the gliomas is the extracellular glutamate concentration maintaining. Glutamate is the major excitatory neurotransmitter that is essential for many processes in the brain and specifically for the excitatory synaptic transmission [9, 10]. Normally, the extracellular glutamate concentration is regulated in the brain by astrocytic excitatory amino-acid transporters (EAATs). They are capable to uptake up to 80% of glutamate, released during the synaptic transmission [11]. Glutamate is converted to glutamine inside astrocytes and released to neurons via X^−^_*c*_—cysteine-glutamate antiporters, which are highly presented in these cell types. This glutamate-glutamine cycle is an important mechanism of the regulation of the pull of neurotransmitter that also prevents overexcitation of neurons nearby. It is well known that reactive astrocytes in the brain tumors have different properties and expression of transporters. Several evidences suggest that expression of EAATs [12], especially EAATs type 2 in reactive astrocytes, is significantly lower than in normal [2]. Additionally, glutamate released from tumor cells leads to neuronal hyperexcitability due to the activation of glutamate receptors on peritumoral neurons and causes an epileptic activity in them [13]. The increased X^−^_*c*_ activity provides an increased cysteine uptake, which is used for glutathione synthesis during an intracellular reduction to cysteine. Glutathione as an intracellular antioxidant is especially important in malignant cells as they neutralize produced reactive oxygen species. Additionally, elevated extracellular glutamate interacts with glutamate receptors (AMPAR and mGluR) on glioma's cells and stimulates cell proliferation, migration, and invasion. Due to the loss of glutamate homeostasis, neurons become susceptible to glutamate-mediated excitotoxicity and die as a result of overstimulation of neuronal glutamate receptors. It leads to uncontrolled rise of intracellular Ca^2+^, aberrant neuronal signaling, and ultimately excitotoxic neuronal death. It also has been shown that reactive gliosis causes a loss of glutamine synthetase, the enzyme involved in the conversion of glutamate to glutamine [14]. In the nonpathological brain, extracellular glutamate concentration is tightly maintained at ~1 mM [12, 13], but outside the synaptic cleft it can be as low as 25 nM [14]. However, it has already been shown that concentration of glutamate in the gliomas culture can increase from 1 *μ*M to 100 *μ*M in 5-6 hours [15].

Therefore, glioma cells alter peritumoral astrocyte EAAT function, promoting the spread of tumors and preventing the reestablishing of glutamate homeostasis, as it would be in the healthy brain.

### 2.2. Glioma Cells Migration

Individual tumor cells or groups of tumor cells can migrate and lead to the development of new tumors. There are at least two targets of cells migration: first the perivascular space and second the brain parenchyma. Both of them usually are associated with blood vessels [16]. Reactive astrocytes can shrink and dramatically change their volume during the time. That happens due to effluxes of chloride ions followed by efflux of water through aquaporin channels (AQPR 1 or AQPR 4). In pathological conditions, activation of NKCCL cotransporters in glioma cells allows them to reach the inside concentration of chloride ions around 100 mM which is 10 times higher than in normal conditions [16]. Since astrocytic processes participate in synaptic transmission and end-feet of astrocytes envelope blood vessels, astrocytes play the role of certain intermediaries between the neuronal activity and regulation of the blood flow. Migration of tumor cells along blood vessels is accompanied with the breaking of matrix components by family of metalloproteinases [17] and leads to the destruction of the connection between astrocytes end-feet and blood vessels that will disrupt the blood-brain barrier and cause the degradation of tight junctions.

Moreover, reactive astrocytes release a huge amount of glutamate (which is also attractant to pathological cells); first cells can serve as pioneers, which go along the vessels and release glutamate as guidance molecule to other following cells.

Thus gliomas cells produce the significant impact on the distraction of the surrounding microenvironment in the result of their ability to change the volume and the significant mobility through the brain [18, 19].

## 3. Intraoperative Diagnostics of Human Glioma Tumors

The treatment of gliomas is a crucial task for modern medicine and represents a complex procedure that includes the microsurgical removal of the tumor, radiotherapy, and chemotherapy, supplemented with photodynamic therapy and immunotherapy (Table 1). However, despite significant progress achieved, the results of treatment of patients with malignant tumors remain unsatisfactory.

### 3.1. Surgical Resection

The implementation of surgical resection remains the major and most crucial step of the treatment. Surgical resection allows the removal of a large amount of tumor tissue, to reduce intracranial hypertension and the degree of neurological deficit, and to establish the exact phenotype of the tumor to choose further treatment strategy. The main challenge of the surgical resection is to remove maximum possible pathological tissue of the tumor and eliminate viable tumor cells from the peritumoral area with minimal functional damage to the surrounding healthy tissue of the brain. However, the total resection of glial tumors is not possible because of the tumors infiltrative growth. Malignant tumor cells can spread into the surrounding tissue up to several centimeters from the primary tumor site. Thus, the remaining tumor cells can serve as a source of continuing tumor growth.

Early studies have been demonstrating that the amount of resection has no significant effect on overall survival and disease-free patients with total and partial removal of the tumor. However, most of them were performed in the period before the development of magnetic resonance tomography (MRI) for patients' monitoring in the postoperative period. Moreover, the volume of the removed tumor had been estimated subjectively during the operation by the surgeon.

Modern studies suggest that the size of the tumor resection significantly correlates with life expectancy of patients [20–22]. The overall survival rate was significantly higher both in patients with the total removal of the tumor and in patients with partial deletion, with a large field of resection compared to patients with partial deletion performed with a small resection. At the present time the amount and degree of resecting high-grade gliomas are estimated by the accumulation of the contrast agent at site T1 on sequences of MRI images. However, a common methodology of treatment for this assessment does not exist. Variation in radical surgery procedures makes the comparison between the results of different authors complicated [22–27]. It introduces considerable difficulties for the final determination of the contrasted tumor volume for the complete resection and for the complete resection of enhancing tumor (CRET) [26].

Removal of the maximum possible amount of the tumor tissue within the physiological permissibility is a key goal of surgical treatment phase. The main constraints to achieve the maximum resection of malignant gliomas (gross total resection) (≥95–98%) are the deficient visualization of tumor boundaries, due to their infiltrative growth, and the necessity of the preserving functionally important areas of the brain.

The traditional removal of the tumor is done under the white-light microscope that has a low resolution; thus, the maximum resection is performed in only 23–50% of cases [21, 28, 29]. This approach required the development of new methods for intraoperative diagnosis of malignant brain tumor boundaries.

Therefore, in order to achieve the maximum resection with minimal risk of the following complications, currently several functional intraoperative techniques have been developed and implemented. They include intraoperative computer tomography (CT) and/or MRI, ultrasonography, neuronavigation, fluorescent diagnostics, intraoperative neurophysiological monitoring, “conscious” craniotomy, and various combinations of these methods [30–32]. These intraoperative diagnostic methods are based on several approaches: the first is the registration of contrast agents, which accumulate in the tumor vasculature (CT, MRI). The second is the registration of metabolic changes in the tissue (special modes of MRI: MR spectroscopy, fluorescent diagnostics, and laser spectroscopy).

The third is the registration of changes in the tissue density (ultrasonography). The exception is the navigation systems, which represent intraoperatively static results of the preoperative examination of patients.

### 3.2. Image Guided Surgery

Neuronavigation or image guided surgery is useful for the precise localization of targets during surgery in a real time. The possibility of direct comparison of the anatomy of the scalp, skull, and brain MRI/CT images allows performing the minimum required size craniotomy. Neuronavigation also helps in determining the most suitable place on the surface of cortex to start resection of the tumor. The accuracy of the method of the tumor boundaries determination depends on the ratio between the static pre-MRI/CT and the location in the brain, which varies considerably during the operation. This brain shift occurs due to the tumor removal and changes in intracranial pressure due to intraoperative injection of hyperosmolar solutions, evacuation, and liquor gravity. This fact makes it impossible to implement this method for the determination of the internal boundaries of the tumor during surgery.

The study of Wirtz et al. [33] suggests that in 52 results of the patient's treatments with glioblastoma the total removal of the tumor has been achieved using neuronavigation in 31% of cases, while in the control group it has been achieved only in 19%. Relative and absolute amount of residual tumor on MRI was also significantly lower in the group with the use of neuronavigation. It should be noted that the time of surgery in both groups did not differ; however, the preparation before neuronavigation usually takes 30.4 minutes. At the same time, a prospective randomized study conducted by Willems et al. showed that the amount of residual tumor in the surgical standard group was 28.9%, and in the group with the navigation it was 13.8%. Mean values of nondeleted tumor volume according to postoperative MRI contrast enhancement were 29.2% and 24.4%, respectively. All the differences were not statistically significant. It is noteworthy that the total removal of the tumor was achieved in five patients in the standard surgery and in three patients with the navigation. The authors concluded that routine use of neuronavigation is inappropriate to define the boundaries of malignant gliomas and achieve a high degree of radicalism. Thus, neuronavigation is a valuable tool for runtime access and early removal of the tumor, but the phenomenon of displacement of the brain in the process of removal of the tumor makes the accuracy of the method insufficient in the determination of the boundaries of malignant gliomas.

### 3.3. Intraoperative Ultrasonography

The intraoperative ultrasonography (iUS) is mostly implemented at the first stage of surgery to visualize the tumor site and surrounding brain structures, including the ventricles, blood vessels, gyrus, and rigid structures such as the falx and tentorium. This method well performs in subcortical gliomas surgery with cystic components [34]. iUS provides images in real time and does not depend on the displacement of brain structures during operation unlike neuronavigation. Currently, for the convenience with the orientation for the surgeon during surgery, there are devices which combine neuronavigation and 3D ultrasound sensors. Availability of iUS allows the surgeon a better navigation in the surgical bed and gives rough information about the presence of residual tumor tissue. In a small series of observations of Unsgaard et al., patients received a standard surgery with subjectively made total removal of the tumor. Then, they used 3D iUS to determine the residual tumor volume, which was detected in 53% of cases [35]. In operations conducted by Gerganov et al., a comparison of the efficacy of intraoperative MRI and iUS without contrast enhancement in diagnosis showed low efficiency in the case of iUS disseminated tumor process and superficial tumor sites [36]. At the same time, using the ultrasound may be implemented in the alternative iMRI deep tumors of small size and for the low-grade gliomas. In general, the low resolution of a standard iUS is not enough to determine the exact boundaries of infiltrative growing of malignant gliomas. A promising method was developed on a base of iUS with the application of the contrast medium (contrast enhanced ultrasound, CEUS), which allows the obtaining of the blood flow characteristics in the tumor tissue. The advantage of this method is the ability to visualize, differentiate, and diagnose the tumor feeding arteries and draining veins with nonrelated vessels feeding the healthy brain tissue. The application of the contrast medium improves the visualization of residual tumor, allowing the increase of the degree of radical surgery [37]. The method is more informative if glial tumors are highly malignant (e.g., glioblastomas) and they have a significantly higher degree of vascularity compared to the surrounding brain tissue. However, the critical point in the using of CEUS contrast agent is that these molecules characterize by the limited ability for diffusion to the vascular bed, accumulate in the interstitial space used in the case of intraoperative MRI agents. Consequently, due to coagulation of vessels feeding the tumor, the value of the method is reduced and contrast agent does not accumulate in the area of the tumor.

Thus, application of ultrasonography may be effective for intraoperative navigation in real time and approximate determination of residual tumor. Due to insufficient resolution of iUS, it does not represent the ideal method for determination of the boundaries of malignant gliomas and achieving total removal of the tumor.

### 3.4. Intraoperative Magnetic Resonance Tomography

The application of the intraoperative MRI with contrast enhancement (iMRI) has a high degree of sensitivity and is considered currently as the “gold standard” in determining the extent of radical removal of the tumor.

The method is to visualize the accumulation of contrast medium on the border of the removed tumor bed on T1-weighted sequences iMRI, allowing determining the presence of residual tumor as contrasted “thin strip” (“thick linear”) and “tumor site” (“tumor-like”), followed by its removal in one operation, which is to be confirmed by histological examination of the tumor contrasted sections [38, 39]. Due to the infiltrative growth of malignant glioma, tumor cells may be present outside of the tumor on the contrast MRI images [40], which is confirmed also by using iMRI, the resolution of which is sufficient (0.5–1 mm range) to distinguish the tumor infiltration and the damage of the brain tissue. The study of Yankeelov et al. has shown that 41–68% of brain tissue biopsy specimens show signs of tumors, despite the lack of accumulation of contrast medium [41]. The overall results of the study confirmed that iMRI is a highly informative method that was evidenced by the measure of the accumulation of a contrast agent in the high-grade tumors (Kendall, correlation coefficient 0.5), and the absence of the tumor correlated with the lack of contrast enhancement.

Most studies reporting promising results of studies on the implementation of iMRI and about the enhancement in the radical surgical interventions with high-grade gliomas nevertheless show a quite low level of efficiency that does not exceed Class B [42]. Only one class of study, Class A, shows a fairly high percentage of maximum resection (96%: iMRI group and 68%: the control group, *p* = 0.023) [43]. At the same time, Kubben et al. using standard neuronavigation have shown a nonsignificant difference between the amount of residual tumor in iMRI group and the control group (13% and 6.5%, resp., *p* = 0.28) [44]. Also, no significant difference was observed in the median survival (396 and 472 days, resp., *p* = 0.81).

Despite the high informational value, iMRI method has several disadvantages, which include a high cost, inability to combine it with a microscope and surgical instruments, and the difficulty of the technical implementation. This leads to a significant increase in duration of the diagnostic procedure. The study of Hirschberg et al. demonstrated that the average time of surgery using iMRI lasts 5.1 hours which is significantly longer than the 3.4 hours of standard operation [45].

### 3.5. Fluorescence Diagnostics

For today the most common approach for the delineating of malignant gliomas is implementation of specific substances—photosensitizers for the fluorescent visualization of the tumor tissue. Their ability to interact with the light and the particular emission wavelength of fluorescence allow the surgeon to determine the tumor and its borders. The effectiveness of 5-aminolevulinic acid (5-ALA) has been shown in a phase III multicenter randomized controlled study of 270 patients [46, 47]. The share of the maximum resection in the study and control groups was 65% and 36%, respectively (difference in efficacy was 29%, *p* < 0.0001). A number of review papers also indicate the effectiveness of fluorescence diagnosis in achieving the maximum amount of resection [48, 49]. The advantages of this method also include the possibility of simultaneous photodynamic therapy and the residual tumor resection of the primary boundary zone. It allows to destroy the tumor structure due to the generation of free radicals or singlet oxygen photosensitizer during the interaction with a more powerful light emission [50]. According to evidence of Russian authors, the fluorescence navigation in the diagnosis of glial tumors Grades I-II is 58.8% and tumors Grades III-IV 89.7% [51]. However, there are certain restrictions on the implementation of the fluorescent neuronavigation. Several researchers indicate varying degrees of fluorescence depending on the degree of malignancy [52, 53], wherein the assessment of the fluorescence is produced by the surgeon and is subjective, which can lead to conservation of a tumor tissue, which has a weak fluorescence signal [54]. The method is capable diagnosing small amounts of photosensitizer accumulated in tumor cells, which are deficient for the induction of fluorescence in a visible laser spectroscopy. The sensitivity of this method for the diagnostics of gliomas is increased to 88% and specificity to 82%. Since a significant obstacle for the quantitative determination of tumor markers is the significant variations in the optical properties of the nervous tissue, depending on the degree of malignancy and associated pathophysiological processes, a combining spectroscopic technique has been developed which takes into account the scattering properties, perfusion, and tissue oxygenation, which expands the diagnostic capabilities. In general, the size of resection using contrasting fluorescence diagnosis greatly exceeds the volume of the tumor compared to preoperative MRI images (84 cm^3^ and 39 cm^3^, respectively, *p* = 0.0087) [55], which should be considered during the surgery in functionally important brain areas.

The main drawback of the method is the variation of fluorescence. Variability in intensity of fluorescence among malignant gliomas can be explained by the presence of differences in the intracellular drug metabolism and/or pharmacokinetics features due to the operation of the blood-brain barrier and cellular transport mechanisms [56]. With a high degree of specificity, fluorescence diagnostics have an insufficient degree of sensitivity and may give false positive results. It is unlikely that the use of photodithazine may lead to overdiagnosis of cancer, but perhaps the reason is the lack of accumulation of contrast agent in areas with a high density of tumor cells [29].

Each tissue type in the body differs from the others and has a specific metabolic rate and degree of its the microcirculation characteristic. Any pathological process in the brain leads to changes in microcirculation and metabolism and, as a result, changes in temperature of the cerebral cortex. Using intraoperatively thermal thermography, it is possible to determine the boundaries of the tumor process based on this method and therefore evaluate the zone of invasion [57–59].

The implementation of these methods, taking into account their characteristics, may lead to significant improvements in resection surgery of glial tumors of high grade. However, due to specific limitations of each method in determining the boundaries of tumor invasion, it remains the actual problem to find new technologies for intraoperation visualization of brain structures in normal and pathological conditions. Current research on this topic includes investigation of the possibility of applying confocal microscopy [60] and optical coherence tomography [61] for these purposes.

## 4. Discussion

We reviewed the wide diversity of modern intraoperative diagnostic methods with its specific advantages and disadvantages. However, the critical disadvantage among all these methods is that total resection of glial tumors is not possible. Thus, the key direction of the development of the modern techniques is the design of efficient diagnostic methods based on currently used approaches, such as magnetic resonance tomography, ultrasonography, neuronavigation, fluorescent diagnostics, intraoperative neurophysiological monitoring, and “conscious” craniotomy in different configurations. Surgical resection serves as the major tool for the removal of the main tumor tissue. However, the full removal of the tumor remains unreachable. Presently, the maximal resection in combination with other tools does not exceed 96% in rear cases and more frequently 88%. Moreover, the high variability of the maximal resection is aggravated by the subjective estimation of the tumor borders by the surgeon. Neuronavigation is characterized by the requirement of the minimum size craniotomy although the result highly depends on the brain motion that also leads to fluctuations of the efficiency. Ultrasonography allows the visualization of the tumor site and surrounding tissue and is not affected by the displacement of brain structures during the operation. On the other hand, ultrasonography does not provide a sufficient resolution. Magnetic resonance tomography shows a significantly high degree of sensitivity and allows determining the presence of residual tumor. The disadvantage of magnetic resonance tomography is the high cost. Additionally, combining of the magnetic resonance tomography with microscopy remains infeasible, and in general, this method is limited by the difficulty of the technical implementation. Fluorescence diagnostics allow defining borders of the tumor, to implement the simultaneous photodynamic therapy and to perform the residual tumor resection of the primary boundary zone. Nevertheless, efficiency of this method depends on variability in the fluorescence intensity and suffers from the low degree of sensitivity. Contrast enhanced ultrasound enables visualizing, differentiating, and diagnosing the tumor feeding arteries and draining veins with nonrelated vessels feeding the healthy brain tissue which provide the incensement of the radical surgery degree. However, the significant concern about this method is the ability of the contrast agent to be accumulated in the interstitial space.

Despite the significant success in the development of intraoperative diagnostic methods for the tumor treatments, existing methods are not sufficient. The following surgery irradiation therapy leads to a significant decline in cognitive functions and causes a memory loss that highlights the importance of the development of alternative approaches for the treatment. The summary of obtained knowledge on physiology of transformed glial cells shall serve as an especially important direction for that purpose. In particular, an understanding of distinct features in physiology between glioma and glial cells may provide a clue for the creation of specific ways of detection of those changes, such as the monitoring of the extracellular glutamate concentration or shifts in the membrane resting potential. An understanding of these cellular or subcellular alterations in cell physiology might provide a significantly higher precision of the identification of the tumor borders. The methods based on this approach could sufficiently improve the survival rate of patients suffering from different glioma tumors and improve the quality of their life. Therefore, the study of gliomas' physiology and the search of new methods of treatments represent a key direction of modern neurobiology.

## Figures and Tables

**Table 1:** Advantages and disadvantages of modern intraoperation diagnostics methods of human gliomas.

Method	Advantages	Disadvantages	Invasiveness	Duration of the operation
Surgical resection	Removes a large amount of tumor tissue	(i) Total resection of glial tumors is not possible (ii) Volume of the removed tumor is estimated subjectively by the surgeon	+	Time of surgery is 3.4 hours

Neuronavigation	Minimum required size craniotomy	Depends on brain motion fluctuations	+	At least 30 min is required before the operation

Ultrasonography	(i) Visualizes the tumor site and surrounding tissues (ii) Does not depend on the displacement of brain structures during operation	Insufficient resolution	−	

Magnetic resonance tomography	(i) High degree of sensitivity (ii) Allows determining the presence of residual tumor	(i) High cost (ii) Inability to combine it with a microscope (iii) The difficulty of the technical implementation	−	Time of surgery is 5.1 hours

Fluorescence diagnostics	(i) Allows determining the tumor and its borders (ii) Possibility of simultaneous photodynamic therapy and the residual tumor resection of the primary boundary zone	(i) Depends on variability in the intensity of fluorescence (ii) Insufficient degree of sensitivity	+	Time of surgery is 3.4 hours

Contrast enhanced ultrasound	(i) Visualizes, differentiates, and diagnoses the tumor feeding arteries and draining veins with nonrelated vessels feeding the healthy brain tissue (ii) Increasing of the degree of radical surgery	(i) Contrast agent accumulates in the interstitial space (ii) Does not work if vessels have been coagulated	−	
